# Evolutionary ecology of aging: time to reconcile field and laboratory research

**DOI:** 10.1002/ece3.2093

**Published:** 2016-03-28

**Authors:** Martin Reichard

**Affiliations:** ^1^ Institute of Vertebrate BiologyAcademy of Sciences of the Czech RepublicBrnoCzech Republic

**Keywords:** Condition‐dependence, evolution of aging, gene‐by‐environment interaction, intrapopulation variability, intraspecific aging rate, mortality, senescence

## Abstract

Aging is an increase in mortality risk with age due to a decline in vital functions. Research on aging has entered an exciting phase. Advances in biogerontology have demonstrated that proximate mechanisms of aging and interventions to modify lifespan are shared among species. In nature, aging patterns have proven more diverse than previously assumed. The paradigm that extrinsic mortality ultimately determines evolution of aging rates has been questioned and there appears to be a mismatch between intra‐ and inter‐specific patterns. The major challenges emerging in evolutionary ecology of aging are a lack of understanding of the complexity in functional senescence under natural conditions and unavailability of estimates of aging rates for matched populations exposed to natural and laboratory conditions. I argue that we need to reconcile laboratory and field‐based approaches to better understand (1) how aging rates (baseline mortality and the rate of increase in mortality with age) vary across populations within a species, (2) how genetic and environmental variation interact to modulate individual expression of aging rates, and (3) how much intraspecific variation in lifespan is attributable to an intrinsic (i.e., nonenvironmental) component. I suggest integration of laboratory and field assays using multiple matched populations of the same species, along with measures of functional declines.

## Introduction

Aging (senescence) is an increase in mortality risk with age due to deterioration of vital functions. Understanding the mechanisms and consequences of aging is not only an intriguing evolutionary question (Jones et al. 2014) but also a matter of practical concern with pressing demographic and societal implications (Fontana et al. 2014). The two aspects of aging research – fundamental understanding of why organisms age and how aging patterns in nature vary on the one hand, and an applied perspective dealing with biomedical treatments of aging on the other – have entered an exciting phase. I argue that the next steps to understand the biology of aging should combine approaches and concepts used by the two research communities.

Biogerontologists are interested in proximate mechanisms of aging, use laboratory models and focus on means of mitigating specific functional declines associated with aging (Gems and Partridge 2013; López‐Otín et al. 2013). This approach provides evidence that longevity of laboratory animals (including natural populations in captivity) can be extended through environmental, dietary, pharmacological and genetic interventions (Partridge and Gems 2007; Lee et al. 2013; López‐Otín et al. 2013). Single gene manipulations are known to extend life in model laboratory animals (Ladiges et al. 2009), with molecular pathways modulating aging shared among species (Fontana et al. 2010). Yet, recent developments demonstrate that fundamental assumptions about the functional basis of aging, such as the concept of oxidative damage (molecular damage by reactive oxygen species arising from metabolic and immune processes) need to be revisited (Selman et al. 2012; Speakman et al. 2015) and new variants of functional concepts, such as the hyperfunction theory (carry‐over activity of early‐life programs for growth and reproduction into later life; Blagosklonny 2008), are gaining increasing support (Gems and Partridge 2013).

Evolutionary biologists seek to understand why aging has evolved and how and why it varies among populations and species. Long‐standing theories to explain the evolution of aging have recently been found unsatisfactory in their ability to explain many observed patterns of aging (Reznick et al. 2004; Chen and Maklakov 2012; Kimber and Chippindale 2013), revealing how incomplete our understanding of the evolutionary aspects of aging (and variation in aging rates within and among species) currently is. While unexpected patterns of aging are usually found in comparisons within species, a large‐scale analysis of aging rates across the tree of life (Jones et al. 2014) emphasized that complexity of aging patterns is much higher than expected and predicted from current theories.

A systematic feedback between functional and evolutionary research on aging is needed to provide mutually beneficial critical insights into the biological basis of aging (Monaghan et al. 2008). The need for mutual feedback is also highly pertinent within the discipline of evolutionary biology, where current practice often tends to focus exclusively on a laboratory (captive)‐based or field‐based approach. While studies using captive‐based and field data in combination (Bronikowski et al. 2002; Kawasaki et al. 2008; Ricklefs 2010b; Hämäläinen et al. 2014) are few, they clearly demonstrate how much lifespan and aging patterns can differ between captive and wild environments. However, data on aging in the wild and in the laboratory are sometimes lumped together without properly acknowledging their distinction (e.g., Jones et al. 2014), thereby undermining the benefits of adopting the two approaches together.

Advantage needs to be taken of the burgeoning methodological advances to estimate functional declines associated with aging provided by biogerontology (e.g., Baumgart et al. 2012; Nussey et al. 2014), many of which can be applied to tackle evolutionarily relevant questions, both in captivity and in natural populations. I propose that a combination of laboratory and field research using matching populations will shed new light on the roles of environmental conditions and gene‐by‐environment interactions on the evolution and expression of aging and age‐related functional declines. In turn, biogerontologists will benefit from insights into the complex relationships between aging, other life‐history traits and ecological setting, which can provide a more general synthesis of specific genotypic and phenotypic alterations.

## Recent Inconsistences in the Evolutionary Theory of Aging

The evolution of aging has long been ascribed to trade‐offs in response to patterns of adult extrinsic mortality arising as a byproduct of selection to maximize reproduction (Medawar 1952; Williams 1957; Kirkwood 1977; Kirkwood and Rose 1991). This trade‐off occurs because mortality from external sources, such as predation or accident (i.e., extrinsic mortality), erodes the strength of natural selection later in life. Under high extrinsic mortality, few individuals survive to reproduce at later ages, leaving selection for longevity irrelevant. This enables accumulation of mutations with deleterious effects that are expressed only later in life (Mutation Accumulation; Medawar 1952), or with age‐specific effects supporting early‐life fitness at the expense of late‐life detrimental effects (Antagonistic Pleitropy; Williams 1957), i.e., favoring investment into early reproduction at the expense of self‐maintenance (Disposable Soma; Kirkwood 1977). As a consequence, higher intrinsic mortality (i.e., more rapid deterioration of vital functions with age) evolves, manifested as rapid aging (increase in mortality rate with age) and limited lifespan, even in the absence of external sources of mortality (i.e., in captivity).

More recent theoretical developments have emphasized greater complexity of basic assumptions, such as the roles of condition‐dependent extrinsic mortality (Williams and Day 2003) and density‐dependence in modulating mortality rates as population density declines (Abrams 1993). Condition‐dependence in extrinsic mortality emphasizes the positive association between individual quality and survival (i.e., survival of the fittest individuals). The standard evolutionary theories of aging (Kirkwood and Austad 2000) and their earlier experimental tests (e.g., Stearns et al. 2000) considered extrinsic mortality as being random with respect to individual quality (condition), with equal likelihood of mortality from extrinsic factors (e.g., predation, disease, inability to cope with periods of challenging climatic conditions) regardless of individual condition and, as an extension, genetic background. Clearly, strong condition‐dependent survival may select for higher physiological performance (e.g., promoting superior ability to escape predator attacks) at the population level. In this sense, condition‐dependent survival can also be viewed as a genetic correlation between stress resistance and lifespan (Rose et al. 1992; Parsons 1995; Holzenberger et al. 2003), with longer lifespan arising as a byproduct of selection on stress resistance. Furthermore, independent of individual condition, density‐dependent effects on survival and fecundity may reverse the effects of extrinsic mortality on evolutionary trajectories of aging by increasing per capita resource availability to survivors (Abrams 1993).

Such added complexity leads to sometimes contrasting predictions and appears to better explain aging in some natural populations (Reznick et al. 2004), at least in captivity. In particular, condition‐dependence in extrinsic mortality will undoubtedly have a considerable effect on the evolution of lifespan, at least within a species. Chen and Maklakov (2012) demonstrated that a longer lifespan evolved in experimental *Caenorhabditis remanei* nematode populations when increased mortality was imposed by heat shock (i.e., condition‐dependent), while a shorter lifespan evolved when mortality was applied at random (i.e., condition‐independent). Given that mortality in the wild typically arises from a combination of condition‐dependent and condition‐independent sources and frequent discrepancies between aging studies in captive and wild populations (e.g., Ricklefs and Cadena 2007; Boonekamp et al. 2014), it remains a challenge to test these predictions across divergent populations under natural conditions.

A recent modeling study (Shokhirev and Johnson 2014) has confirmed that the underlying assumptions of extrinsic mortality have a fundamental impact on its effect on evolutionary trajectories of aging. This approach indicated that high extrinsic mortality favored a longer lifespan when the cost of reproduction was high and resources were not limiting. In contrast, high extrinsic mortality was predicted to select for dramatically shorter lifespan when resources were scarce and reproduction was not costly. Such scenarios may be grossly approximated to K and r‐ selected life histories (Pianka 1970), represented, for example, by a large herbivore and a small opportunistic rodent, or to equilibrium and periodic fish life histories (Winemiller and Rose 1992). In this context (with some simplification), high extrinsic mortality would select for longer lifespan in elephants but for shorter lifespan in lemmings. Longer and shorter lifespans were accompanied by increased and decreased investment into self‐maintenance, respectively, suggestive of an impact on intrinsic mortality rate (Shokhirev and Johnson 2014). This conceptual framework may also help elucidate sexual differences in aging patterns, where females pay a higher cost of reproduction (Maklakov and Lummaa 2013), and provides predictions that are particularly amendable to experimental testing.

## We Need Measures of Functional Decline in the Wild

**Figure 1:**
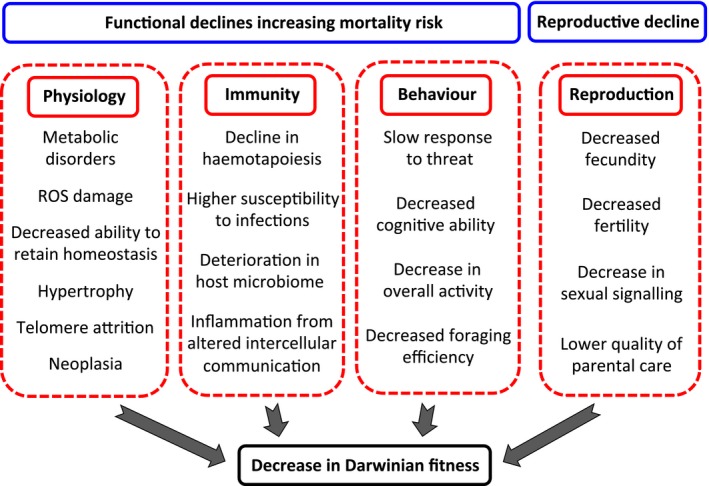
Components of aging.

A multitude of functional declines are associated with the aging process (Fig. 1), which may or may not be correlated (Massot et al. 2011; Hayward et al. 2013). The demography of aging (actuarial aging), a principal focus of field studies, should be supplemented with insights into the proximate factors leading to an increase in mortality with age. Functional declines are associated with degradation of physiological functions, decay in immune response or a decreasing ability to maintain homeostasis under the challenges of metabolic activity (López‐Otín et al. 2013). Detrimental products of metabolic activity can be directly quantified in a variety of tissues and recent advances in genetic and genomic resources permit even more direct approaches, such as longitudinal studies of gene expression in metabolic pathways (Pincus et al. 2011). At the organismal level, functional declines such as decrease in cognitive ability, reduction in activity pattern or slower response to threat, can also be tested in behavioral assays (Reznick et al. 2004; Terzibasi et al. 2008). Notably, reproductive senescence emphasizes the important fact that the reproductive value (fertility, fecundity, parental care) of an individual can decline without associated changes in mortality (Coulson et al. 2006), and this is where most effort in field‐based studies has so far been concentrated (e.g., Robinson et al. 2012; Boonekamp et al. 2014).

Nine hallmarks of aging (genomic instability, telomere attrition, epigenetic alterations, loss of proteostasis, deregulated nutrient sensing, mitochondrial dysfunction, cellular senescence, stem cell exhaustion, and altered intercellular communication), that represent common denominators of aging in different organisms, have recently been defined (López‐Otín et al. 2013) and I think these should be targeted to test their effects across study taxa. In the “‐omics” era, minute tissue samples can potentially deliver complex information. If a study animal can be easily and repeatedly captured, blood or skin samples can provide information on its physical and physiological condition, immune function or gene expression levels. This would represent a major development from current practice where individual vigor is typically estimated indirectly (e.g., through courtship displays, competitive interactions or overall activity patterns). Longitudinal studies can relate information on specific individuals observed across their lifespan (Lemaître et al. 2014).

At the population level, age‐related changes in these parameters can provide valuable information on the dynamics of functional traits responsible for aging. With the knowledge that condition‐dependence is a crucial aspect of individual variation in survival (Williams and Day 2003; Chen and Maklakov 2012; Hämäläinen et al. 2014), however, we should aim primarily at longitudinal, individual‐based measures to provide a foundation for understanding which traits provide animals with condition‐dependent survival. Functional aspects of aging are processes unique to an individual and the onset and slope of age‐dependent declines are expected to vary even among individuals within a population (Roach and Carey 2014). Such individual‐based studies are common in biomedical research involving humans but are surprisingly rare for animals, even under laboratory conditions (Fontana et al. 2014). Longitudinal data on allocation to reproduction, either as mating effort or number of progeny, have provided useful composite measures of functional declines in many bird and mammal species (e.g., Nussey et al. 2008; Robinson et al. 2012; Santos and Nakagawa 2012; Boonekamp et al. 2014) and there is no question that the approach can be used for other functional aspects of aging and other organisms in natural populations. In addition to longitudinal assays of functional traits that provide individuals with condition‐dependent survival probability (e.g., muscular function enabling a startle response), studies should ideally be complemented with estimates of aging biomarkers deposited as end‐products of metabolic activity, with apparently less direct effect on condition‐dependent survival (e.g., deposits of lipofuscin in various tissues; Terzibasi Tozzini et al. 2013). This would provide a control between estimates of biomarkers of chronological age and actual functional aging.

Recent work has moved toward this approach, demonstrating that even robust component traits, such as the ability to regain body mass after a seasonal decline, can represent a powerful surrogate for functional deterioration (Hämäläinen et al. 2014). Direct analysis of physiological traits, immunocompetence or gene expression levels, however, should enable more direct, mechanistic measures of the processes associated with aging in nature. Thus, major insights into aging can be achieved by linking demographic approaches with measures of functional deterioration at the behavioral, physiological, cellular and reproductive levels in wild populations.

## Aging in the Context of Life History Trade‐Offs

The complex nature of the aging process, and the selective forces acting on longevity, are underpinned by evolutionary trade‐offs between aging and other life‐history traits. Life‐history theory predicts that, at a high risk of extrinsic mortality, lifetime fitness is generally maximized by rapid growth, early maturation and high allocation to reproduction at the expense of neglecting self‐maintenance (Kirkwood and Rose 1991; Stearns 1992). At the interspecific level, age at first reproduction in wild populations of birds and mammals is closely and negatively related with the onset of a decline in survival and reproduction (Jones et al. 2008; Ricklefs 2010a), as predicted by the theory. In contrast, at the intraspecific level, the opposite pattern emerges (Charmantier et al. 2006; Robinson et al. 2012), perhaps because condition‐dependent survival does not predict trade‐offs between life history traits. Our understanding of whether the rate at which different organisms age (i.e., the slope of increase in mortality rate with age) follows the same evolutionary trend as the onset of a decline is currently poor. Interestingly, a recent study demonstrated that the shape of mortality curve was conservative among lifespan‐affecting treatments in *Caenorhabditis elegans* and that intraspecific lifespan differences were primarily driven by a simple temporal scaling of mortality risk (Stroustrup et al. 2016). While this is an extremely promising discovery, the relationship between extrinsic mortality and aging may not be so straightforward and further challenges will inevitably arise regarding our understanding of how aging is associated with other life‐history traits, especially at the intraspecific level.

## Gene‐By‐Environment Interactions

Genes and environment strongly interact to modulate aging patterns (Kenyon 2010; de Magalhães et al. 2012), though little is known about the relationship between aging in a benign laboratory environment compared with that in nature. Gene‐by‐environment interaction is regularly overlooked in aging research on animal models studied under laboratory conditions (Partridge and Gems 2007). Environmental context, however, alters longevity and performance, even in inbred strains of laboratory model animals (Nussey et al. 2013). Individual, population, or species‐level genetic background will impinge on the expression of aging traits under contrasting environmental conditions (Bronikowski et al. 2002; Reznick et al. 2004; Williams et al. 2006; Hämäläinen et al. 2014). Environmental context may modulate individual ability to cope with challenges presented by metabolic waste products (Selman et al. 2012; Speakman et al. 2015), reproductive investment (Nussey et al. 2013) or other intrinsic sources leading to stronger functional deterioration. Ultimately, this leads to condition‐dependent survival (Darwin 1859; Williams and Day 2003; Chen and Maklakov 2012).

To understand the evolution of aging it is crucial that we recognize how the natural environment and natural selection interact to shape aging rates and associated life‐history trade‐offs. The availability of pedigree information from several natural populations of birds and mammals (e.g., Charmantier et al. 2006; Wilson et al. 2007) has enabled quantitative genetic estimation of aging in the wild under complex environmental challenges (Wilson et al. 2008). Furthermore, common garden experiments using natural and laboratory populations provide a powerful tool to test interactions between experimentally selected environmental factors and defined genetic background on aging (Reznick et al. 2004). While these approaches help us comprehend interactions between environmental and genetic effects, there is a lack of direct feedback between studies from the field and those under controlled conditions (Ricklefs 2008). This is not surprising since combining estimates of mortality in the wild and in the laboratory is impractical for most taxa. Researchers typically utilize one of several approaches, depending on their study system and immediate goals (Table 1). While all these particular avenues of research are informative, it remains challenging to integrate their respective advantages within a single model system. This would provide insights that the individual approaches could not deliver.

**Table 1:** Current approaches in aging research, the insights they provide and their advantages and limitations

Name	Taxa	Insights	Advantages	Limitations
Targeted genetic modifications	Established laboratory models (yeast, *Drosophila, C. elegans*, mice), wider range with recent development of CRISPR‐Cas (1)	Biochemical pathways and molecular targets for drug development (e.g., TOR and rapamycin (2))	Opportunity to test the effects of single‐gene manipulations against a fixed genetic background	A fixed genetic background can have profound effects on the phenotypic outcome of a given intervention (3), but see also (4,5) for the use of diverse genetic background)
Comparative genomics of long‐lived animals	Naked mole rats, bluefin whale and other long‐lived species	Genomic variations related to cellular mechanisms that facilitate protection against aging‐related declines (6)	Identification of shared genomic associations with long lifespan	Difficulty of disentangling longevity from other unusual species characteristics, such as eusociality or adaptation to subterranean life
Cross‐sectional analyses of survival in wild populations	Mammals, birds, dragonflies	Specific challenges important to patterns of mortality under natural conditions (e.g., elevated risk of predation or bouts of mortality under particularly challenging environmental conditions) (7,8)	Clear identification of evolutionarily relevant sources of mortality and their timing, and estimates of gene‐by‐environment interactions	Low (if any) replication across populations, comparisons often made at the individual level within a single population (9,10) or between closely related species (8)
Transcriptional and genetic association studies	Humans	Significant general association of APOE and FOXOA3 gene polymorphisms with long life (11); large population‐specificity in other aging‐related polymorphisms (12)	Large‐scale longitudinal data in replicated natural populations, often including details on functional declines	Insight into proximate mechanisms, but not directly into the evolution of aging
Experimental evolution	Short‐lived laboratory animals *(Callosobruchus, Caenorhabditis*,* Drosophila*)	Demonstrating the capacity of specific organisms to respond to selection favoring increased or decreased rates of aging (13,14)	Maintains associated trade‐offs in other life‐history traits	Commonly excludes tests of trade‐offs in response to challenging environment (but see (14)); lab‐adapted populations difficult to associate with natural settings; limited to short‐lived nonvertebrates
Common garden experiments	Various taxa that can be kept in captivity	Revealing genetically‐determined interpopulation variation in aging traits	Standardization of environmental hazards; use of replicated natural populations	More complicated designs are needed to exclude population‐specific adaptations matching specific lab conditions (e.g., ambient temperature)

References: (1) Harel et al. (2015); (2) López‐Otín et al. (2013); (3) Liao et al. (2010); (4) Harrison et al. (2009); (5) Lind et al. (2016); (6) Fang et al. (2014); (7) Hayward et al. (2011); (8) Wilson et al. (2007); (9) Massot et al. (2011); (10) Sharp and Clutton‐Brock (2011); (11) Deelen et al. (2011); (12) Beekman et al. (2013); (13) Stearns et al. (2000); (14) Chen and Maklakov (2012).

## Intraspecific Differences in Aging Rates

**Figure 2:**
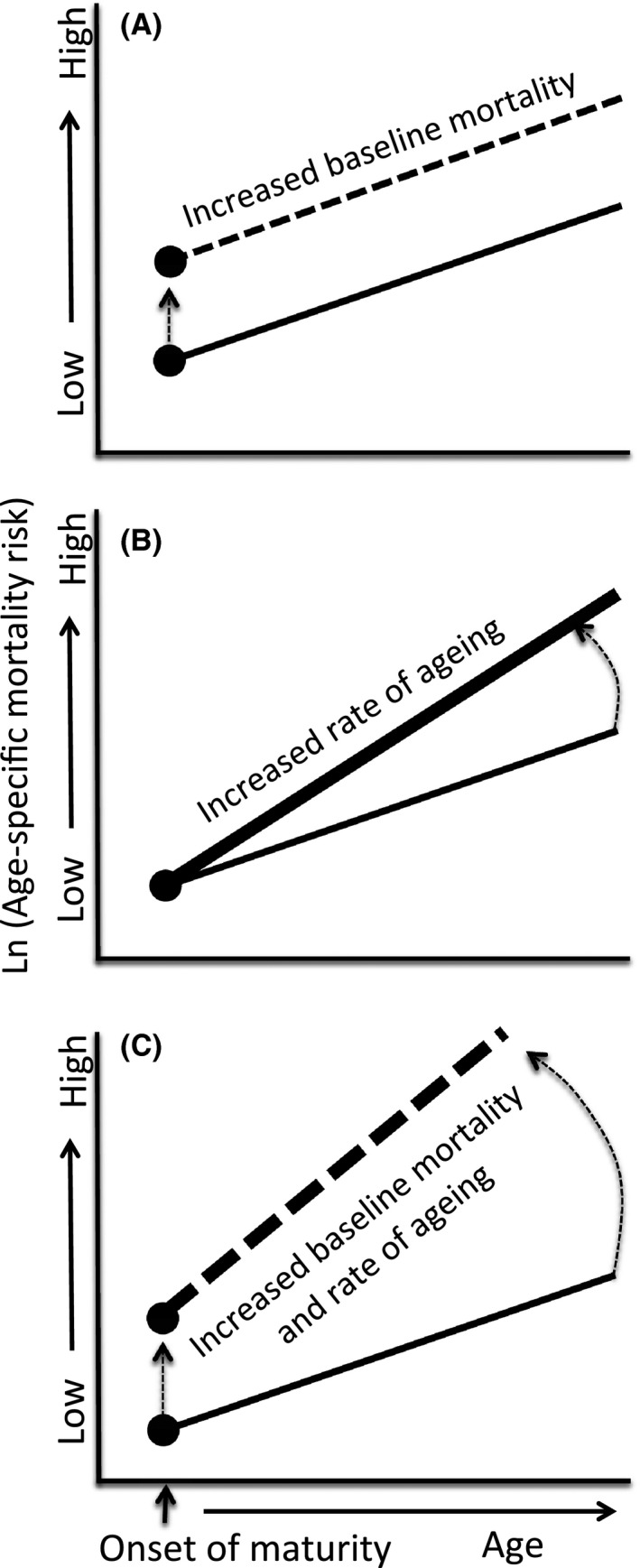
Partitioning variation in mortality. Schematic illustration of how different components of aging can modify adult lifespan, with special reference to the effect of intrinsic and extrinsic mortality. Populations can differ in the rate of aging (i.e., slope of mortality risk increase with age) (A); in the level of baseline (age‐independent) mortality, suggestive of the extrinsic component of mortality (intercept) (B); or a combination of both (C), suggesting that both components of mortality can vary in concert.

**Figure 3:**
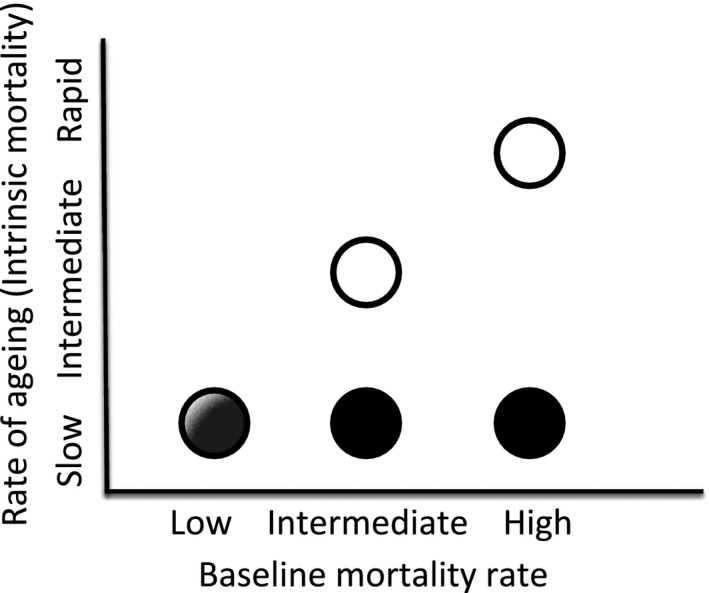
At the micro‐evolutionary scale, rate of aging (age‐dependent, intrinsic mortality) can be unresponsive to changes in baseline (age‐independent) mortality arising from external forces (black circles), or increase along with the elevation in baseline mortality (open circles), as predicted at macro‐evolutionary levels.

While it is well‐recognized that lifespan varies greatly among species (Ricklefs 2010a; Jones et al. 2014), there is also considerable intraspecific variation (Bronikowski et al. 2002; Kraus et al. 2013; Lohr et al. 2014), suggesting that natural selection can substantially and rapidly modify this trait within species. Lifespan (i.e., longevity) and aging rate (i.e., rate of increase in mortality with age), however, are often not correlated (Baudisch 2011). Theoretically, a short‐lived population may experience low aging – a high background mortality rate leads to short lifespan despite a negligible increase in mortality with age (and hence negligible aging). In contrast, a long‐lived population with low background mortality rate can experience rapid increase in mortality rate at the old age and hence undergo a high aging rate despite its long lifespan. At present, we have too little data to understand interpopulation variability of aging in natural populations (but see Beekman et al. 2013 for human populations). The few nonhuman studies available (Bronikowski et al. 2002; Reznick et al. 2004; Morbey et al. 2005) suggest that intraspecific differences in lifespan in natural populations may be derived from variation in baseline mortality (Bronikowski et al. 2002; Morbey et al. 2005; Fig. 2A), which is inherently strongly dependent on environmental factors, including population density (Pletcher et al. 2000). In contrast, an increase in mortality risk with age (age‐specific mortality, a measure of intrinsic mortality; Pletcher et al. 2000) is relatively similar across populations of the same species (Bronikowski et al. 2002; Reznick et al. 2004; Morbey et al. 2005) or among closely related species (Terzibasi Tozzini et al. 2013). Indeed, Ricklefs (2010b), using a nested comparative approach, has demonstrated that variation in actuarial senescence appears phylogenetically constrained and that most variation is detected at the level of order and family, with little variation among closely related genera and species. Does this mean that intrinsic aging rates are relatively conserved across populations for a given species, or even a higher taxon? Can intrinsic aging respond to natural selection rapidly, at a micro‐evolutionary scale such as the outcome of eco‐evolutionary feedbacks (Fig. 3)?

Current insights into how natural selection can affect aging at the intraspecific level come from studies on experimental evolution and from artificial selection. Experimental evolution is a powerful tool for investigating how standing genetic variation in a population responds to experimental manipulation of conditions, including the risk of extrinsic mortality. For example, by applying either random or condition‐dependent mortality, Chen and Maklakov (2012) were able to select for increased or decreased lifespan in a population of nematode worms, *C. remanei*. This and other studies (e.g., Stearns et al. 2000; Maklakov et al. 2007) have demonstrated that there is enough intrapopulation genetic variability to respond to selection on lifespan and aging. However, it is unclear how this rapid response to contrasting conditions can be translated into the multifaceted environment outside the laboratory. This is pertinent not only to complex trade‐offs with other life history traits under natural environmental challenges that cannot be replicated in captivity or gene‐by‐environment interactions but also other subtleties, such as fluctuations in population density, effects of environmental seasonality or changes in resource levels (Kawecki et al. 2012). Their effects on the strength of aging rate and its evolution may ultimately mask any strong effect of an experimentally directed selection regime.

Domestic breeds can be considered as artificial surrogates for populations with different adaptive optima. Intriguingly, variation in lifespan among dog breeds is primarily due to differences in intrinsic aging rate (i.e., a more rapid increase in mortality with age) rather than baseline mortality (Kraus et al. 2013). Larger dog breeds, therefore, not only live shorter lives, but also age more quickly (Fig. 2B). Larger breeds also suffer a higher incidence of diseases related to hypertrophy and hyperplasia, providing a potential link to the hypertrophy theory of aging (Blagosklonny 2008). Artificial selection, however, may be more similar to experimental evolution than natural selection. Feral populations relaxed from directed artificial selection often rapidly reconstitute their natural phenotype (Sol 2008), indicating that trade‐offs with other traits are of paramount importance. A study on natural populations, where traits are being selected within life‐history trade‐offs and constraints under challenging ecological settings, is therefore needed, to elucidate interpopulation differences in intrinsic aging rates.

A promising approach for separating aging rates into their extrinsic and intrinsic components is to compare how the same genotypes age in unprotected conditions in the wild and a protected laboratory environment (Ricklefs 2010b). In the wild, interpopulation variation in the extrinsic component of mortality is predicted, but would the intrinsic component also covary with environmental conditions when the importance of trade‐offs is not restricted by a benign environment? This approach would ideally be completed in a series of natural populations, where different lifespan or mortality risk is indicative of selection for different aging rates. To date, laboratory and field trials have not been satisfactorily combined in this way (Table 2).

**Table 2:** Overview of research questions, underlying hypotheses, approaches and their challenges, and potential solutions for the research agenda suggested in the paper

Question	Hypothesis	Approach	Challenges	Solutions
How does demographic (actuarial) aging vary across populations within a species?	Is variation of lifespan across populations of a species attributable to differences in (i) background (nonaging) mortality, (ii) steepness of increase in mortality rate with age (aging rate), (iii) onset of age‐related increase in mortality or (iv) their combination?	Collating life‐time survival data on replicated wild populations with a sample size attributable to fitting demographic models (n > 50), ideally with variable (and measurable) levels of extrinsic mortality	Long‐term research program or using short‐lived organisms amendable to individual marking	New advances in bioinformatics now allow for much easier use of mark‐recapture data, providing robust estimates of aging‐related demography (1)
How much intraspecific variation in lifespan is attributable to an intrinsic (i.e., nonenvironmental) component?	Is interpopulation variation in lifespan lower in captivity than in the wild?	Comparing survival data from wild populations with wild‐derived (matched genetic background) populations in captivity	Choice of taxon amendable to field‐ and laboratory‐based estimates (small philopatric animals)	(i) New advances in bioinformatics now allow for easier use of mark‐recapture data (1); (ii) some datasets from the wild can be matched with existing data on captive populations, e.g., in zoos (2)
Are functional declines in the wild comparable to those observed in captivity?	Individuals in the wild experience faster physiological deterioration due to more challenging conditions (stronger stress)	Estimating identical functional declines in wild and captive (wild‐derived) populations with a matched genetic background	Nondestructive sampling to enable collection of longitudinal (individual‐based) dataset	High performance methods using minute tissue samples from blood, feathers, epidermal or hair samples (hormonal assays, immunosenescence, oxidative stress, telomere attrition) (3–5)
How much variation of longevity within a natural population is attributable to gene‐by‐environment interactions?	Environmental context significantly modulates the expression of aging rates	Common garden experiment with split‐clutch (family) design manipulating key stressors. Alternatively, a cross‐fostering experiment in the wild (or semi‐natural setting) across contrasting social or environmental conditions	Long‐term research agenda for most study taxa	Using short‐lived species for common garden experiments (6,7), taxa with low dispersal/high recapture rates (8) or seminatural cross‐fostering experiments ((9)

References: (1) Colchero et al. (2012); (2) Ricklefs and Cadena (2007); (3) Nussey et al. (2014); (4) Schneeberger et al. (2014); (5) Wilkening et al. (2016); (6) Reznick et al. (2004); (7) Terzibasi Tozzini et al. (2013); (8) Hämäläinen et al. (2014); (9) Boonekamp et al. (2014).

## Challenges in Combining Field and Laboratory Assays

The main challenge to combining research in the wild and the laboratory using matching populations is that animals amenable to lifetime observation in the wild are generally not amenable to rearing in captivity (and vice versa) due to their size, lifespan or environmental requirements. The overriding advantage of studying populations in the wild is that environmental variables induce evolutionarily relevant condition‐dependent survival and generate trade‐offs between different components of life history (Nussey et al. 2013). Studying aging in the wild, however, presents several major difficulties. First, natural populations typically have a diffuse structure, leading to unsatisfactory recapture rates (Kawasaki et al. 2008). Second, overlapping age classes yield heterogeneity in the environmental conditions experienced by the study individuals (e.g., Bryant and Reznick 2004; Nussey et al. 2007; Hayward et al. 2009; Hämäläinen et al. 2014). Third, current field studies provide data on mortality and reproductive senescence but give limited opportunity for assays on other functional declines, or at least sources of mortality (e.g., Bonduriansky and Brassil 2002; Bronikowski et al. 2002, 2011; Santos and Nakagawa 2012).

A very limited subset of organisms has been used to study aging in both the laboratory and the wild. Trinidadian guppies (*Poecilia reticulata*) from high and low predation risk populations have contrasting life expectancy in the wild (Bryant and Reznick 2004) but display reversed lifespans under protected laboratory conditions (Reznick et al. 2004). Guppies are theoretically amenable to combining a laboratory approach with field estimates of mortality, aging patterns and functional declines in a single comparative study. The wealth of background information on their natural history (Evans et al. 2011), their short lifespan in the wild and small body size predispose the species for this work. The guppy model, however, illustrates many of the challenges to be overcome. Guppies are short lived in the wild (<1 year) but captive guppies may live up to 4 years (Reznick et al. 2004), making such assays impractical. Wild guppies can be individually marked and recaptured reliably (Bryant and Reznick 2004) but reproduce year‐round and different individuals are subject to different demographic and environmental conditions (Arendt et al. 2014), rendering standard analysis of aging rates problematic.

Annual killifishes, with their much shorter lifespan (<1 year in captivity), restricted dispersal and single, nonoverlapping age cohorts may represent a more suitable taxon for such comparative work (Cellerino et al. 2016). They inhabit temporary savanna pools and have evolved naturally short lifespans as a response to annual desiccation of their habitats. The fish hatch from desiccation‐resistant eggs when their habitat is filled with water (Polačik et al. 2011) and achieve sexual maturity in a few weeks (Blažek et al. 2013). Their synchronous hatching ensures that all individuals in a population are challenged by the same environmental conditions. Adult fish die when their habitat desiccates but their lifespan is temporally condensed rather than prematurely terminated and, at least in the laboratory, they suffer from a range of functional declines (Valenzano et al. 2006; Di Cicco et al. 2011) and selective mortality is observed in the wild (Reichard et al. 2014). Killifish populations form discrete units (Bartáková et al. 2015) and are large enough to remove individuals for analyses of functional declines without affecting demography. Their size makes individual marking possible while the requirements for laboratory breeding are not demanding (Polačik et al. 2016). A study of their aging in the wild, however, has not been accomplished. Other taxa, despite their longer lifespan, may also be suitable to combine field and laboratory aging assays (e.g., lizards: Massot et al. 2011).

To my knowledge, there has been just one study, using a single population of a neriid fly, which has combined estimates of aging in the wild and under laboratory conditions on the same natural population. Kawasaki et al. (2008) followed almost a thousand individually marked *Telostylinus angusticollis*, a fly associated with rotting trees, and showed that survival rates in the wild were five‐times lower than those under protected conditions in a laboratory environment. While this pioneering study was illuminating, several drawbacks may have masked important insights. For example, adult flies were laboratory‐reared and continuously released over an extended period of 3 months. Given that their life expectancy was 3–4 days, changes in environmental conditions could potentially have generated seasonal variation, confounding the findings and decreasing the power to quantify and interpret aging rates. Furthermore, only actuarial aging was studied, with functional responses at the physiological, histological, gene expression and reproductive level untested.

Further challenges for studies on matched wild and captive populations include maternal and epigenetic effects that have the potential to affect laboratory assays. Maternal effects arising from differential allocation of maternal provisioning among offspring, or from the environment experienced by the mother early in life, can potentially introduce a confounding component into common garden conditions, creating false variation ascribed to genetic background; though this could be mitigated by experimental design (e.g., Reznick et al. 2004). As an extension, epigenetic effects can potentially affect life histories for multiple generations (Dias and Ressler 2014) and can be even more difficult to control. Research on epigenetic effects is a burgeoning research field and new insights will increase our understanding of their impact on longevity and aging rates at both the population and individual levels.

## Current Insights from Interpopulation Comparisons

Two studies have directly compared outbred captive primate populations with wild, unmanaged populations of the same species. Both confirmed that the environment strongly modifies life expectancy due to higher extrinsic (baseline) mortality in more challenging conditions (Bronikowski et al. 2002; Hämäläinen et al. 2014). Importantly, a study on one captive and two wild baboon (*Papio hamadryas*) populations suggested that intrinsic mortality may be fixed at the species level (Bronikowski et al. 2002). The other study, which compared wild and captive populations of mouse lemur (*Microcebus murinus*), revealed that gross estimates of functional decline (the capacity to regain seasonally variable body mass) may be difficult to detect in the wild due to strongly selective, condition‐dependent survival (Hämäläinen et al. 2014). This highlights the importance of carefully choosing the measure of functional decline as wild populations, despite experiencing clear aging, have much shorter lives. Meta‐analyses of human aging studies, which effectively compare several natural populations, further suggest that aging is a trait with large interpopulation differences (Beekman et al. 2013).

Further insights can be gleaned from specific contrasts between natural and laboratory conditions. For example, a striking bias in adult sex ratios is widespread in wild populations (Arendt et al. 2014; Reichard et al. 2014), despite matching populations showing no sex bias over their captive lifespans. It appears that sexual selection can elevate mortality and weaken selection on male lifespan (Bonduriansky et al. 2008), thereby generating an intralocus sexual conflict (Berg and Maklakov 2012). Intersexual differences in aging are one particularly promising research avenue for understanding the costs and benefits of aging in relation to reproductive investment (Chen and Maklakov 2014; Cornwallis et al. 2014).

## Conclusions

The main challenges emerging in research into evolutionary ecology of aging are a lack of understanding of functional senescence under natural conditions and the role of gene‐by‐environment interactions in modulating aging rates at the intraspecific level. A key goal is to integrate data from wild and laboratory assays by encompassing demographic approaches (actuarial aging) with organismal deterioration at the behavioral, physiological, cellular and reproductive levels. I believe that substantial insights into aging will be obtained by comparing aging rates in matched populations exposed to natural and laboratory conditions, using individuals from replicated populations within species exposed to contrasting selection regimes (such as different risks of mortality). This approach will allow us to fully elucidate the role of gene‐by‐environment interactions on condition‐dependent survival and intraspecific variation in aging rates.

## Conflict of Interest

None declared.
